# Analysis of the common bean (*Phaseolus vulgaris* L.) transcriptome regarding efficiency of phosphorus use

**DOI:** 10.1371/journal.pone.0210428

**Published:** 2019-01-18

**Authors:** Daiana Alves da Silva, Siu Mui Tsai, Alisson Fernando Chiorato, Sónia Cristina da Silva Andrade, José Antonio de Fatima Esteves, Gustavo Henrique Recchia, Sérgio Augusto Morais Carbonell

**Affiliations:** 1 Instituto Agronômico (IAC)–Centro de Grãos e Fibras—Fazenda Santa Elis, Campinas, SP, Brazil; 2 Centro de Energia Nuclear na Agricultura (CENA)–Av. Centenário, São Dimas–CEP–Piracicaba, SP, Brazil; 3 Universidade de São Paulo (USP)—Departamento de Genética e Biologia Evolutiva–Instituto de Biociências–Rua do Matão, Cidade Universitária–Cep–São Paulo, SP, Brazil; INRA, FRANCE

## Abstract

Common bean is a highly important food in tropical regions, where most production occurs on small farms with limited use of technology and, consequently, greater vulnerability to abiotic stresses such as nutritional stress. Usually phosphorus (P) is the most limiting nutrient for crop growth in these regions. The aim of this study was to characterize the gene expression profiles of the genotypes of common bean IAC Imperador (P-responsive) and DOR 364 (P-unresponsive) under different P concentrations using RNA-seq transcriptome sequencing technology. Plants were grown hydroponically, with application of two P concentrations (4.00 mg L^-1^ restrictive level and 8.00 mg L^-1^ control level). Differential expression analyses, annotation, and functional classification were performed comparing genotypes within each P rate administered and comparing each genotype response to the different P levels. Considering differential expression analyses within genotypes, IAC Imperador exhibited 1538 up-regulated genes under P restriction and 1679 up-regulated genes in the control, while DOR 364 exhibited 13 up-regulated genes in the control and only 2 up-regulated genes under P restriction, strongly corroborating P-unresponsiveness of this genotype. Genes related to phosphorus restriction were identified among the differentially expressed genes, including transcription factors such as WRKY, ERF, and MYB families, phosphatase related genes such as pyrophosphatase, acid phosphatase, and purple acid phosphatase, and phosphate transporters. The enrichment test for the P restriction treatment showed 123 enriched gene ontologies (GO) for IAC Imperador, while DOR 364 enriched only 24. Also, the enriched GO correlated with P metabolism, compound metabolic processes containing phosphate, nucleoside phosphate binding, phosphorylation, and also response to stresses. Thus, this study proved to be informative to phosphorus limitation in common bean showing global changes at transcript level.

## Introduction

Most common bean (*Phaseolus vulgaris* L.) production in developing countries occurs on small farms with limited technology, resulting in vulnerability to pest attack and abiotic stress, including water deficit and low soil fertility. Thus, development of new, higher-yielding cultivars bred for resistance to biotic and abiotic stresses is the main objective of plant breeding programs around the world [1]

Crop yield depends on the nutrients available in the soil solution and on nutrient uptake by the plant. Different nutrients are structurally or functionally involved in the plant life cycle and are essential for plant growth and reproduction [2] Phosphorus (P) is considered the most limiting nutrient for growth of legume crops in tropical and subtropical regions and, since it is a non-renewable resource, successive crops result in continual degradation of soil P in the absence of additional fertilization [3] (Due to the interaction of inorganic phosphorus (Pi) with other elements, up to 80% of Pi applied can be fixed in the soil, forcing farmers to apply up to four times more than necessary for production.

Thus, increasing the efficiency of P use by crops has been a challenge, largely due to the complex interactions among the range of acquisition mechanisms and their effectiveness in different environments. MOURICE & TRYPHONE (2012) [4] also emphasize the importance of the use of bean varieties that are able to acquire phosphorus in environments with limiting soils, and this ability is attributed to genetic variability. Therefore, the study of gene relationships that determine greater efficiency of specific genotypes to acquisition and use of the nutrient is important.

According to GEORGE & RICHARDSON (2008) [5], plants have a wide range of physiological mechanisms and characteristics that facilitate an increase in the availability and acquisition of P from the soil. It is known that P restriction rapidly reduces rates of plant growth. Phosphorus deficiency alters cell biochemistry, biomass allocation, and root morphology to meet plant P demands. Typical responses of plants to P restriction include remobilization, reduction, or substitution of P in non-essential cell compounds, exudation of metabolites and enzymes to the rhizosphere, and changes in root morphology. There may be associations with microorganisms to increase the efficiency of P acquisition from the soil [6]Nevertheless, P demand in plant tissues and responses in regard to availability of the element vary among genotypes. SILVA et al. (2014) [7] assessed 20 common bean genotype responses to P nutritional deficiency in hydroponics through evaluation of morphophysiological and agronomic traits such as leaf chlorophyll content, shoot and root dry matter, leaf area, root area, root diameter, root length, and root volume, as well grain yield components Five genotypes stood out and were classified as efficient and responsive to P use: IAPAR 81, Carioca Comum, IAC Carioca Tybatã, IAC Imperador, and G 2333, whereas DOR 364 was one of the most inefficient and unresponsive genotypes. According to FAGERIA et al. (2015) [8], ER characterizes plants that have high grain yield at low P availability and are responsive to phosphate fertilization, whereas NENR indicates plants that have low yield at low P levels and low response to phosphate fertilization.

HERNÁNDEZ et al. (2007) [9], studying the functional genomics of common bean plants under reduction in P supply, obtained the expression profile of the root system of the genotype Negro Jamapa 81 through macroarray analyses. They identified 126 differentially expressed genes with diverse functions in plant adaptation to P deficiency, contributing information on regulation and on signaling pathways during deficiency of the nutrient.

The aim of this study was to evaluate the agronomic traits and the gene expression profiles of the root system regarding P use efficiency in two contrasting common bean genotypes, IAC Imperador and DOR 364, grown hydroponically, and compare their response to application of a restrictive P concentration, 4.00 mg L^-1^, and a control P concentration, 8.00 mg L^-1^. RNA-seq analysis was used to detect differences in gene expression between genotypes and P application rates. Correlations between the function of differentially regulated genes and the physiological traits of both bean genotypes are discussed in light of potential applications for P management in bean crops.

## Results

### Analysis of agronomic traits

At 41 days after transplanting, the flowering stage (R6) was reached, and plants of both genotypes were collected for biometric analyses. All the shoot parameters (LA, LDM, SDM, PH, NNP) exhibited highly significant statistical differences (P>0.01) in relation to the P application rate factor (Table 1). The greater availability of P provided by the control treatment resulted in an increase in all the parameters, except for plant height (Table 2). In relation to the genotype factor, a statistical difference could be seen only for leaf dry matter (Table 1)—IAC Imperador exhibited 54.22% more leaf dry matter production than DOR 364, namely, mean values of 11.61 g for IAC Imperador and 6.29 g for DOR 364 (Table 2). In addition, a significant difference was also observed for the P application rate vs genotype interaction, likewise for the LDM variable (Table 1).

**Table 1:** Summary of analysis of variance of the traits related to the shoots, root system, yield components, and grain yield of the IAC Imperador and DOR 364 common bean genotypes grown hydroponically under a restrictive and control phosphorus level.

**SV**	**DF**	**Mean square—Shoots**				
		LA	LDM	SDM	PH	NNP
**P rate**	1	56650111**	72.39**	114.56**	8164**	8.333**
**Genotypes**	1	2109247	84.85**	9.13	61	0.000
**P rate x Genotypes**	1	931304	15.52*	14.83	252	0.333
**Residue**	8	1861100	2.00	3.02	493	0.500
**CV%**		13.60	15.81	11.99	15.67	4.61
**SV**	**DF**	**Mean square—Root system**				
		RL	RSA	RV	RD	RDM
**P rate**	1	65853831	279200	1.3	0.00121	1.26
**Genotypes**	1	389862011**	4831724**	370.1**	0.0000002	6.98**
**P rate x Genotypes**	1	27381	578	0.3	0.000046	0.17
**Residue**	8	15442839	261722	30.9	0.0003814	0.33
**CV%**		11.2	12.9	15.37	5.44	18.78
**SV**	**DF**	**Mean square–Yield components**				
		NP	NS	NSP	100SW	GY
**P rate**	1	520	22360	0.6724	1.44	2408*
**Genotypes**	1	3710**	128961**	0.0559	39.5**	13967**
**P rate x Genotypes**	1	2	147	0.128	8.08*	227
**Residue**	8	130	4879	0.6057	1.14	387
**CV%**		29.71	30.23	13.18	3.69	28.67

*,**Significant at 0.05 and 0.01 by the F test.

**Table 2:** Mean performance of the IAC Imperador and DOR 364 common bean genotypes grown hydroponically under a control and restrictive phosphorus condition in regard to shoot, root, yield component, and yield traits. Scott-Knott test <0.05.

**SV**	**LA** (cm^2^)	** **	**LDM** (g)	** **	**SDM** (g)	** **	**PH** (cm)	** **	**NNP**	** **
					
Control	12202.83	a	11.41	a	17.56	a	167.83	a	16.16	a
Restrictive P rate	7857.33	b	6.49	b	11.39	b	115.66	b	14.5	b
IAC Imperador	1044.93	a	11.61	a	15.34	a	144	a	15.33	a
DOR 364	9610.83	a	6.29	a	13.6	a	139.5	a	15.33	a
**SV**	**RL** (cm)	** **	**RSA** (cm^2^)	** **	**RV** (cm^3^)	** **	**RD** (mm)	** **	**RDM** (g)	
					
Control	37413.06	a	4124.47	a	36.48	a	0.369	a	3.372	a
Restrictive P rate	32727.84	a	3819.41	a	35.81	a	0.348	a	2.725	a
IAC Imperador	40770.31	a	4606.48	a	41.69	a	0.359	a	3.81	a
DOR 364	29370.58	b	3337.39	b	30.59	b	0.358	a	2.285	b
**SV**	**NPP**	** **	**NSP**	** **	**NSP**	** **	**100SW**	** **	**GY** (g)	
					
Control	45	a	274.16	a	6.14	a	29.2	a	82.76	a
Restrictive P rate	31.83	a	187.83	a	5.66	a	28.51	a	54.43	b
IAC Imperador	56	a	334.66	a	5.97	a	30.66	a	102.71	a
DOR 364	20.83	b	127.33	b	5.83	a	27.04	b	34.48	b

In relation to the root system traits, a significant difference was not observed for the P application rate factor. In relation to the genotype factor, significant differences (P>0.01) were observed (Table 1). IAC Imperador had 27.97% greater root length (RL), 27.55% greater root surface area (RSA), 26.64% greater root volume (RV), and 40.15% greater root dry matter (RDM) than the DOR 364 genotype (Table 2).

In relation to yield components, analyses of variances showed statistical difference for the phosphorus application rate factor only for the GY. For the genotype factor, highly significant differences (P>0.01) could be seen for all the traits evaluated (NP, NS, 100SW, and GY), except for NSP. There was a significant effect for the P application rate vs genotype interaction only for the 100SW trait (Table 1).

Although the P application rate supplied did not result in statistical differences for the yield component traits (NP, NS, NSP, and 100SW), it resulted in a 41.37% greater increase for NP, 45.96% for NS, 8.48% for NSP, and 2.42% for 100SW in the control P rate than in the restricted P rate. The superiority of IAC Imperador was also clear as it had 62.8, 61.97, 2.34, 11.84, and 66.19% better performances than DOR 364 for the NP, NS, NSP, 100SW, and GY traits, respectively (Table 2).

### Uptake efficiency, translocation, and P use indexes

The mean concentrations of P in the leaves, branches, roots, and grain in both treatments were assessed in order to calculate the indexes of nutrient uptake and use (Table 3). Mean concentrations in each tissue were higher in the treatment that received the control application rate.

**Table 3:** Mean P concentration (g.kg^-1^) in the leaves, branches, roots, and grains of the IAC Imperador and DOR 364 common bean genotypes grown hydroponically under a restrictive phosphorus condition and under a control condition.

Genotype	Application rate	Leaves	Branches	Roots	Grain
		g.kg^-1^ P			
DOR 364	Restricted	2.293	2.003	0.513	3.790
IAC Imperador	Restricted	2.363	1.503	0.353	2.767
Mean		2.328	1.753	0.433	3.2785
DOR 364	Control	3.403	3.220	0.633	4.307
IAC Imperador	Control	3.097	1.943	0.567	3.663
Mean		3.250	2.582	0.600	3.985

The P uptake and use efficiency indexes were calculated and subjected to analyses of variance, showing significant effects for all the traits in relation to the P application rate treatment. In relation to the genotype factor, only the P translocation efficiency index did not show a significant difference. The coefficients of experimental variation had satisfactory values, ranging from 26.52% for the uptake efficiency characteristic to 0.66% for the P translocation efficiency characteristic, which had better experimental effectiveness (Table 4).

**Table 4:** Summary of analysis of variance in regard to the P uptake and use efficiency indexes of the IAC Imperador and DOR 364 common bean genotypes grown hydroponically under a restrictive phosphorus condition and under a control condition.

SV	DF	Mean Square–P uptake and use efficiency indexes					
		PUE	PTE	PUES	PUET	PUEGP	PHI
**P rate**	1	1283.3**	0.0010274**	0.07074**	0.12117**	0.907*	62.75**
**Genotypes**	1	238.3*	0.0000757	0.02124**	0.0357**	4.474**	17.54*
**P rate x Genotypes**	1	33.1	0.0000009	0.0012	0.00003	0.465	3.07
**Residue**	8	34.1	0.0000413	0.00126	0.00199	0.133	1.96
**CV%**		26.52	0.66	8.17	9.22	28.5	19.16

*,**Significant at 0.05 and 0.01 by F test.

In regard to the P uptake efficiency index (PUE), significant differences were observed between the P treatments applied, showing that the greater the supply of the nutrient, the greater its uptake; thus, the control treatment (index of 32.34) showed greater uptake of the element than the P restriction treatment (index of 11.66). DOR 364 showed greater efficiency in uptake of the element, with an index of 26.46, while IAC Imperador had an index of 17.54.

In regard to the translocation efficiency index (PTE), there was a statistical difference at 1% probability only for the P application rate factor, with indexes of 0.98 and 0.96 for the control and restrictive P level, respectively. The genotypes showed very similar values for this index, and it was not possible to detect a difference in translocation between them. This index showed the lowest coefficient of environmental variation (0.66%), which shows high experimental effectiveness for this trait.

The efficiency index of P use in the shoots (PUES) showed greater use of the element in the restricted P application rate, which was 31.45% greater than in the control rate, a highly significant difference. In relation to the genotypes, IAC Imperador showed a better P use index for the shoots, which was 0.48; whereas DOR 364 exhibited an index of 0.39.

A similar result was observed for the total P use efficiency index (PUET); there was greater total use of the element by the plants that received the restricted application rate, 34.48% more than the control rate. IAC Imperador proved to be more efficient in the use of total P, with an index of 0.53, while DOR 364 exhibited an index of 0.43.

Analysis of variance in regard to the efficiency index for P use in the shoots for grain formation (PUEGP) showed statistical difference for P application rates and for genotype. The treatment that received the restricted P rate showed a greater PUEGP index, 1.55, whereas the control treatment had an index of 1.01. IAC Imperador, higher yielding in both treatments, had an index of 1.89, almost three times greater than the DOR 364 index, which was 0.66.

For the P harvest index (PHI), a highly significant difference was observed for the P rates applied, and the treatments with the restricted and control P rates had indexes of 9.59 and 5.01, respectively. This result implies greater accumulation of P in the grains of the stressed treatments, that is, in the treatments in which lower grain yield was observed. There was also a significant difference for the genotypes; DOR 364 had a higher P harvest index, 8.51, while the P harvest index of the more productive genotype, IAC Imperador, was 6.09.

### RNA-seq data

Sequencing of the libraries generated approximately 450 million paired reads. After filtering the low quality reads, the total number dropped to approximately 400 million sequences, from which 207 million reads were from the libraries that received P restriction (Samples 1–6), and 193 million were obtained from the control treatment (Samples 7–12). The 12 libraries data set were published in a public repository (NCBI) in SRA accession: PRJNA498535 (https://www.ncbi.nlm.nih.gov/sra/PRJNA498535).

Approximately 396 million reads were mapped on the common bean genome used as a reference (Pvulgaris 218 v1.0), from the Phytozome v.9.1 database, with an average of 99.2% of mapped reads. From a total of 373 million reads that were mapped in only one region, 193 million were observed in the treatment with P restriction and 180 million in the control condition.

### Differential expression analysis

#### Comparison1. IAC Imperador vs. DOR 364 at the restrictive P level

**Fig 1:**
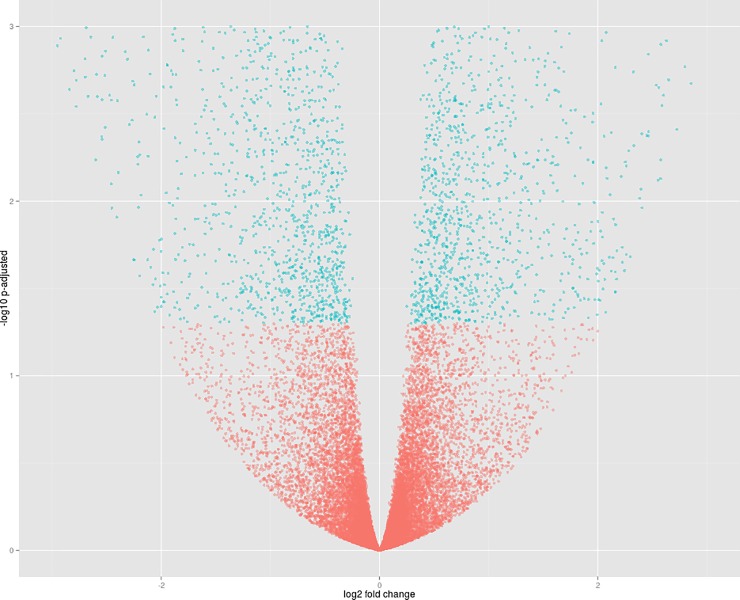
Volcano plot of comparison 1. Dots in blue represent significant differentially expressed genes. The genes of the IAC Imperador genotype (Libraries 1–3) are represented to the left of the origin of the coordinate plane, and the genes of DOR 364 (Libraries 4–6) to the right.

Considering the six libraries that received the P restriction treatment, 30,060 expressed genes were identified, of which 27,191 are annotated according to the common bean unigenes. After FDR correction through the Benjamini-Hochberg procedure and baseMean filtering, 24,632 genes with unique sequences were identified, of which 4,123 showed significant differential expression levels (16.72%). In this condition, IAC Imperador showed 2,159 overexpressed genes, whereas DOR 364 had 1,964 (Fig 1).

Among the genes differentially expressed under P restriction, 48 transcription factor related genes were up-regulated in IAC Imperador and another 53 were up-regulated in DOR 364 (Table 5). Notably, the majority of these transcriptional regulator genes were identified as the WRKY family (12 up-regulated in IAC Imperador and 3 up-regulated in DOR 364). Ten up-regulated ethylene-responsive transcription factors were also found in IAC Imperador and four in DOR 364 (Table 5). Another five up-regulated transcription factors were identified from the MYB family, one in IAC Imperador and 4 in DOR 364 (Table 5).

**Table 5:** Up-regulated transcription factor unigenes in IAC Imperador and DOR 364 under phosphorus restriction.

	Unigene	Brief description	Up-regulated	baseMean	log2FoldChange	lfcSE	padj
1	Phvul.008G256900	Wrky transcription factor 12-related	IAC Imperador	343.3265	-1.4449	0.3879	0.0021
2	Phvul.002G019100	Heat stress transcription factor b-1	IAC Imperador	3321.7889	-1.4217	0.3150	0.0001
3	Phvul.001G224600	Transcription factor bhlh149	IAC Imperador	67.3983	-1.0471	0.3486	0.0198
4	Phvul.007G273000	Ethylene-responsive transcription factor 1b	IAC Imperador	735.1155	-3.3564	0.3288	0.0000
5	Phvul.007G128100	Ethylene-responsive transcription factor erf096	IAC Imperador	19.3451	-2.0696	0.6851	0.0189
6	Phvul.002G228400	Heat shock transcription factor	IAC Imperador	246.7498	-2.1952	0.4086	0.0000
7	Phvul.009G262200	Ethylene-responsive transcription factor erf071-related	IAC Imperador	3497.6679	-0.3170	0.1168	0.0414
8	Phvul.002G035100	Ethylene-responsive transcription factor erf019-related	IAC Imperador	57.5909	-1.3775	0.5209	0.0490
9	Phvul.008G000500	Lipopolysaccharide-induced transcription factor regulating tumor necrosis factor alpha	IAC Imperador	984.5052	-0.3683	0.1056	0.0046
10	Phvul.001G088200	Wrky transcription factor 45-related	IAC Imperador	1056.0698	-2.0934	0.2203	0.0000
11	Phvul.001G037000	Heat stress transcription factor a-2-related	IAC Imperador	28.6550	-1.1446	0.4210	0.0411
12	Phvul.008G098300	Trihelix transcription factor gt-3a-related	IAC Imperador	205.7337	-0.9927	0.2587	0.0014
13	Phvul.001G042200	Wrky transcription factor 40-related	IAC Imperador	198.0408	-1.7385	0.2283	0.0000
14	Phvul.003G151300	Bhlh transcription factor	IAC Imperador	490.6153	-0.8339	0.2248	0.0022
15	Phvul.006G119100	Wrky transcription factor 48-related	IAC Imperador	456.3653	-1.5613	0.2000	0.0000
16	Phvul.002G155300	Heat stress transcription factor b-2b	IAC Imperador	985.0829	-0.5228	0.1556	0.0069
17	Phvul.001G131000	Heat shock transcription factor	IAC Imperador	1038.3017	-1.9738	0.2700	0.0000
18	Phvul.003G290600	X-box transcription factor-related	IAC Imperador	422.9239	-1.0105	0.3547	0.0298
19	Phvul.001G160400	Ethylene-responsive transcription factor erf096	IAC Imperador	91.8777	-1.8137	0.6348	0.0291
20	Phvul.010G158300	Bhlhzip transcription factor BIGMAX	IAC Imperador	47.9809	-0.9118	0.3242	0.0326
21	Phvul.002G211200	Transcription factor sac51-related	IAC Imperador	2142.6702	-0.5383	0.1383	0.0012
22	Phvul.009G123300	Ethylene-responsive transcription factor erf014	IAC Imperador	420.6142	-0.8920	0.3122	0.0291
23	Phvul.002G091100	Wrky transcription factor 3-related	IAC Imperador	2215.4433	-0.7292	0.1558	0.0000
24	Phvul.007G127800	Ethylene-responsive transcription factor 1b	IAC Imperador	1081.0761	-1.2212	0.2906	0.0004
25	Phvul.006G074600	Wrky transcription factor 1-related	IAC Imperador	1780.2287	-1.4086	0.2628	0.0000
26	Phvul.001G202800	Thyroid transcription factor 1-associated protein 26	IAC Imperador	1214.5706	-0.6313	0.1637	0.0013
27	Phvul.002G148200	Wrky transcription factor 12-related	IAC Imperador	48.6750	-2.6464	0.5954	0.0001
28	Phvul.005G169100	Transcription factor TMF, TATA element modulatory	IAC Imperador	4182.4597	-0.4369	0.0997	0.0002
29	Phvul.001G160200	Ethylene-responsive transcription factor 1b	IAC Imperador	165.2077	-2.9210	0.5968	0.0000
30	Phvul.006G058700	X-box transcription factor-related	IAC Imperador	119.3885	-0.7681	0.2836	0.0421
31	Phvul.002G290000	Transcription factor egl1-related	IAC Imperador	3517.7084	-1.0308	0.1430	0.0000
32	Phvul.010G114900	Ethylene-responsive transcription factor erf023	IAC Imperador	44.8959	-0.9823	0.3258	0.0192
33	Phvul.001G160100	Ethylene-responsive transcription factor erf096	IAC Imperador	536.2918	-2.7391	0.3358	0.0000
34	Phvul.002G196800	Wrky transcription factor 28-related	IAC Imperador	207.4859	-1.2114	0.3530	0.0056
35	Phvul.003G116300	Wrky transcription factor 48-related	IAC Imperador	409.0692	-1.6410	0.3300	0.0000
36	Phvul.001G009000	Basic-leucine zipper (bzip) transcription factor family protein	IAC Imperador	4974.6775	-0.4560	0.1636	0.0347
37	Phvul.011G205900	Transcription factor GT-2 and related proteins contains trihelix DNA-binding/SANT domain	IAC Imperador	335.6476	-0.9106	0.1740	0.0000
38	Phvul.008G270400	Transcription factor iiia	IAC Imperador	2014.8546	-0.5532	0.1363	0.0006
39	Phvul.009G026900	Transcription factor tga3-related	IAC Imperador	179.0279	-0.8233	0.2775	0.0219
40	Phvul.008G251700	Wrky transcription factor 12-related	IAC Imperador	65.0694	-1.5599	0.3414	0.0001
41	Phvul.008G251800	Wrky transcription factor 12-related	IAC Imperador	930.5593	-3.1689	0.3883	0.0000
42	Phvul.003G244000	Heat stress transcription factor b-1	IAC Imperador	2508.4282	-0.8922	0.1334	0.0000
43	Phvul.005G001000	X-box transcription factor-related	IAC Imperador	4236.7881	-1.3504	0.1818	0.0000
44	Phvul.002G137100	Transcription factor jumonji (jmj) family protein / zinc finger (c5hc2 type) family protein",	IAC Imperador	3058.3200	-1.6355	0.1387	0.0000
45	Phvul.003G062500	Homeobox protein transcription factors	IAC Imperador	43.7426	-1.4070	0.4249	0.0080
46	Phvul.009G043200	Wrky transcription factor 38-related	IAC Imperador	131.0528	-1.3938	0.2651	0.0000
47	Phvul.002G322700	Heat stress transcription factor a-8	IAC Imperador	642.2030	-0.8444	0.1840	0.0001
48	Phvul.003G190400	Myb family transcription factor	IAC Imperador	5.6252	-1.9231	0.7172	0.0449
49	Phvul.003G222900	Myb transcription factor	DOR 364	3491.1499	0.5749	0.1753	0.0089
50	Phvul.004G124300	Transcription factor bhlh118 related	DOR 364	163.2729	0.7444	0.2377	0.0138
51	Phvul.004G057800	Myb transcription factor	DOR 364	104.8247	1.0832	0.3580	0.0186
52	Phvul.003G103500	Nuclear transcription factor y subunit b 5	DOR 364	15.2151	1.8295	0.5667	0.0104
53	Phvul.004G079200	Transcription factor gata gata binding factor	DOR 364	189.0584	1.0387	0.3051	0.0061
54	Phvul.001G196800	Nuclear transcription factor y subunit a 10 related	DOR 364	10892.5883	0.7209	0.2099	0.0055
55	Phvul.007G257100	Transcription factor bhlh117 related	DOR 364	388.9308	0.9346	0.2041	0.0001
56	Phvul.002G215500	Mads box transcription factor anr1	DOR 364	32.5937	2.0061	0.4242	0.0000
57	Phvul.009G138600	Wrky transcription factor 13 related	DOR 364	19.2212	1.7093	0.4790	0.0036
58	Phvul.010G098500	Transcription factor myc1	DOR 364	41.2866	1.3070	0.4205	0.0147
59	Phvul.002G237300	Shn shine	DOR 364	12.6004	1.7135	0.5500	0.0144
60	Phvul.007G100300	Transcription factor bhlh111	DOR 364	3481.1975	0.5604	0.2068	0.0420
61	Phvul.008G172200	Ethylene responsive transcription factor crf5 related	DOR 364	4465.1597	0.3778	0.1406	0.0443
62	Phvul.011G153000	Transcription factor bhlh123	DOR 364	1758.4471	0.5783	0.2068	0.0339
63	Phvul.009G075000	Transcription factor rax2	DOR 364	909.9920	0.9203	0.1724	0.0000
64	Phvul.005G040000	Transcription factor HEX	DOR 364	408.4442	0.6705	0.2518	0.0469
65	Phvul.008G279600	X box transcription factor related	DOR 364	277.8819	1.5242	0.1653	0.0000
66	Phvul.004G124200	Transcription factor bhlh118 related	DOR 364	67.6364	1.7581	0.3683	0.0000
67	Phvul.008G234300	Transcription factor bhlh83 related	DOR 364	72.5192	1.4472	0.4365	0.0079
68	Phvul.005G156100	Nuclear transcription factor y subunit a 3 related	DOR 364	248.7126	1.1025	0.3020	0.0027
69	Phvul.008G196800	Transcription factor pif4 related	DOR 364	33.1095	1.9058	0.4543	0.0004
70	Phvul.008G159400	Shn shine	DOR 364	32.7202	1.6431	0.4343	0.0017
71	Phvul.008G160400	Transcription factor bhlh83 related	DOR 364	282.5604	8.1987	0.5647	0.0000
72	Phvul.002G162500	Shn shine	DOR 364	75.2261	1.0441	0.2890	0.0031
73	Phvul.007G064300	Transcription factor bhlh118 related	DOR 364	242.7131	1.2773	0.1672	0.0000
74	Phvul.001G249300	Transcription factor tga1 related	DOR 364	6620.3923	0.6175	0.1662	0.0022
75	Phvul.002G266400	Wrky transcription factor 13 related	DOR 364	50.2655	1.1687	0.3501	0.0074
76	Phvul.011G099100	Homeobox protein transcription factors	DOR 364	441.2703	0.7260	0.2067	0.0043
77	Phvul.005G105200	Ethylene responsive transcription factor crf5 related	DOR 364	431.7052	1.4220	0.3190	0.0001
78	Phvul.005G119400	Transcription factor lhw	DOR 364	2829.7438	0.6563	0.2001	0.0089
79	Phvul.005G005800	Wrky transcription factor 2 related	DOR 364	1489.1706	0.2898	0.1041	0.0349
80	Phvul.007G015000	Transcription factor tcp21 related	DOR 364	926.2252	0.6039	0.1847	0.0092
81	Phvul.008G279800	X box transcription factor related	DOR 364	656.1761	2.5348	0.2092	0.0000
82	Phvul.002G110500	Transcription factor bhlh49	DOR 364	1205.6837	0.3266	0.1027	0.0120
83	Phvul.003G231200	Transcription factor nai1	DOR 364	265.4907	1.2317	0.3760	0.0090
84	Phvul.011G086200	Transcription factor fer like iron deficiency induced transcription factor	DOR 364	1021.7054	1.3179	0.2246	0.0000
85	Phvul.L008300	X box transcription factor related	DOR 364	2482.8589	0.9407	0.1112	0.0000
86	Phvul.011G018500	PLATZ transcription factor (PLATZ)	DOR 364	1508.4708	0.6030	0.1934	0.0143
87	Phvul.003G094700	Transcription factor bee 3	DOR 364	34.1318	2.3897	0.4573	0.0000
88	Phvul.008G212100	Homeobox protein transcription factors	DOR 364	6.6024	2.0198	0.7313	0.0370
89	Phvul.001G254000	Transcription factor HEX	DOR 364	11936.5647	0.6126	0.2057	0.0212
90	Phvul.007G139300	Myb family transcription factor	DOR 364	858.1583	0.6031	0.1078	0.0000
91	Phvul.008G039300	Shn shine	DOR 364	57.8846	1.6115	0.3787	0.0003
92	Phvul.007G211800	Transcription factor myb108	DOR 364	93.6382	1.5009	0.4571	0.0088
93	Phvul.007G165100	Nuclear transcription factor y subunit b 5	DOR 364	253.5611	1.8406	0.3809	0.0000
94	Phvul.009G245800	Transcription factor gte1	DOR 364	21.2691	2.0580	0.5853	0.0042
95	Phvul.011G098900	Transcription factor lhw	DOR 364	7798.5829	0.5254	0.1280	0.0005
96	Phvul.004G163300	Heat stress transcription factor c 1	DOR 364	219.2929	1.8125	0.3708	0.0000
97	Phvul.011G058000	Ap2 like ethylene responsive transcription factor plt2	DOR 364	171.3930	1.2464	0.3475	0.0034
98	Phvul.008G279700	X box transcription factor	DOR 364	1768.9610	1.0241	0.1605	0.0000
99	Phvul.002G295700	Shn shine	DOR 364	51.8213	0.9428	0.3565	0.0490
100	Phvul.003G222600	Ethylene responsive transcription factor erf035	DOR 364	64.4599	1.0199	0.2783	0.0026
101	Phvul.005G097200	Transcription factor tcp10 related	DOR 364	130.2055	0.8558	0.2838	0.0192

In our analysis, 53 up-regulated phosphatase related genes were found; 32 of them were over expressed in IAC Imperador and 19 in DOR 364. Among these genes, four of them code for pyrophosphatase; six of them code for acid phosphatase, which is known to catalyze the cleavage of inorganic phosphate from a broad array of phosphomonoesters and anhydrides; and two of them code for purple acid phosphatase (Table 6).

**Table 6:** Up-regulated phosphatase related unigenes in IAC Imperador and DOR 364 under phosphorus restriction.

N	Unigene	Brief description	Up-regulated	baseMean	log2FoldChange	lfcSE	padj
1	Phvul.011G148400	Pap-specific phosphatase hal2-like	IAC Imperador	131.579	-1.129	0.288	0.001
2	Phvul.005G111700	Geranyl diphosphate diphosphatase / Geranyl pyrophosphate pyrophosphatase	IAC Imperador	33.312	-3.633	0.630	0.000
3	Phvul.002G304600	Phosphoethanolamine/phosphocholine phosphatase / PHOSPHO1	IAC Imperador	485.028	-0.741	0.165	0.000
4	Phvul.009G031400	Protein phosphatase 2c 10-related	IAC Imperador	854.421	-1.018	0.237	0.000
5	Phvul.003G162700	Peroxisomal nadh pyrophosphatase nudt12	IAC Imperador	870.286	-0.416	0.129	0.011
6	Phvul.008G155300	Phosphoglucan phosphatase lsf1	IAC Imperador	273.202	-0.598	0.167	0.003
7	Phvul.010G121500	Phosphatase dcr2-related	IAC Imperador	7221.152	-1.732	0.254	0.000
8	Phvul.007G032600	Lipid phosphate phosphatase 1-related	IAC Imperador	1014.117	-0.564	0.162	0.005
9	Phvul.006G019300	Protein phosphatase 1	IAC Imperador	35.649	-5.131	0.647	0.000
10	Phvul.006G136400	Phosphoglycolate phosphatase 2	IAC Imperador	406.282	-0.409	0.152	0.044
11	Phvul.005G008300	Trehalose-phosphate phosphatase f-related	IAC Imperador	3666.289	-0.958	0.250	0.001
12	Phvul.010G140700	Phosphatase	IAC Imperador	54.444	-1.746	0.350	0.000
13	Phvul.001G164000	Had superfamily subfamily iiib acid phosphatase	IAC Imperador	716.480	-0.536	0.184	0.025
14	Phvul.010G024200	Protein phosphatase 1	IAC Imperador	789.045	-1.099	0.203	0.000
15	Phvul.005G160800	Phosphoethanolamine/phosphocholine phosphatase / PHOSPHO1	IAC Imperador	789.045	-1.099	0.203	0.000
16	Phvul.008G054300	Atypical dual-specificity phosphatase	IAC Imperador	3604.408	-1.310	0.342	0.001
17	Phvul.006G142400	Inositol-phosphate phosphatase / Myo-inositol-1-phosphatase	IAC Imperador	882.437	-0.771	0.208	0.002
18	Phvul.007G032700	Lipid phosphate phosphatase 1-related	IAC Imperador	569.215	-0.428	0.145	0.022
19	Phvul.007G249800	Inactive purple acid phosphatase 16-related	IAC Imperador	304.010	-6.105	0.506	0.000
20	Phvul.002G005300	Type i inositol 1 5-trisphosphate 5-phosphatase 2"	IAC Imperador	4996.094	-0.346	0.130	0.046
21	Phvul.004G032300	Acylphosphatase / Acylphosphate phosphohydrolase	IAC Imperador	128.949	-1.270	0.330	0.001
22	Phvul.005G111500	Geranyl diphosphate diphosphatase / Geranyl pyrophosphate pyrophosphatase	IAC Imperador	64.612	-2.214	0.651	0.006
23	Phvul.007G259900	Protein phosphatase 2c 68-related	IAC Imperador	2956.936	-0.376	0.109	0.005
24	Phvul.006G139300	ADP-ribose diphosphatase / ADPR-ppase // NAD(+) diphosphatase / NADP pyrophosphatase // Mn(2+)-dependent ADP-ribose/CDP-alcohol diphosphatase / Mn(2+)-dependent ADP-ribose/CDP-alcohol pyrophosphatase	IAC Imperador	294.258	-0.850	0.190	0.000
25	Phvul.006G206300	Protein phosphatase 2c 10-related	IAC Imperador	2022.077	-0.387	0.132	0.024
26	Phvul.011G008700	Purple acid phosphatase 10	IAC Imperador	4254.308	-1.748	0.440	0.001
27	Phvul.005G085100	Serine/threonine protein phosphatase	IAC Imperador	412.709	-0.662	0.175	0.002
28	Phvul.001G258400	Serine/threonine protein phosphatase 2a 55 kda regulatory subunit b' delta isoform	IAC Imperador	1953.997	-0.481	0.137	0.004
29	Phvul.001G259700	Acid phosphatase/vanadium-dependent haloperoxidase-related protein	IAC Imperador	1343.743	-0.559	0.206	0.041
30	Phvul.002G309100	Protein phosphatase 2c	IAC Imperador	212.812	-0.775	0.251	0.016
31	Phvul.001G255800	Dual specificity protein phosphatase	IAC Imperador	4458.404	-0.692	0.260	0.047
32	Phvul.007G118300	Atypical dual-specificity phosphatase	IAC Imperador	40.199	-1.893	0.588	0.011
33	Phvul.003G033100	Bis(5'-nucleosyl)-tetraphosphatase (asymmetrical) / Dinucleosidetetraphosphatase (asymmetrical)"	DOR 364	1201.171	0.335	0.123	0.040
34	Phvul.001G033800	Protein phosphatase 2c 61-related	DOR 364	6127.422	0.922	0.305	0.019
35	Phvul.001G121600	Protein-serine/threonine phosphatase / Serine/threonine specific protein phosphatase // Protein-tyrosine-phosphatase / ptpase"	DOR 364	1602.470	0.686	0.174	0.001
36	Phvul.001G240200	Sedoheptulose-bisphosphatase / Sedoheptulose-1/7-bisphosphatase	DOR 364	10.416	2.990	0.674	0.000
37	Phvul.008G097800	Phosphatidylinositol-3/ 4-bisphosphate 4-phosphatase / Phosphoinositide 4-phosphatase"	DOR 364	2163.082	0.526	0.103	0.000
38	Phvul.002G072400	Alpha-trehalose-phosphate synthase (UDP-forming) / UDP-glucose—glucose-phosphate glucosyltransferase // Trehalose-phosphatase / Trehalose 6-phosphate phosphatase"	DOR 364	2314.244	0.439	0.105	0.000
39	Phvul.002G072400	Acid phosphatase / Phosphomonoesterase	DOR 364	2314.244	0.439	0.105	0.000
40	Phvul.001G240100	Acid phosphatase / Phosphomonoesterase	DOR 364	253.735	0.880	0.225	0.001
41	Phvul.007G199100	Protein phosphatase 2c 5-related	DOR 364	3595.032	0.889	0.149	0.000
42	Phvul.002G324900	Histidine kinase / Protein kinase (histidine) // Protein-serine/threonine phosphatase / Serine/threonine specific protein phosphatase"	DOR 364	4265.770	1.428	0.120	0.000
43	Phvul.010G014800	Pyridoxal phosphatase / Vitamin B6-phosphate phosphatase	DOR 364	1339.503	0.720	0.209	0.005
44	Phvul.003G015500	Histidine kinase / Protein kinase (histidine) // Protein-serine/threonine phosphatase / Serine/threonine specific protein phosphatase"	DOR 364	1309.533	1.111	0.177	0.000
45	Phvul.008G259800	Protein phosphatase 2c-like protein-related"	DOR 364	3313.335	1.134	0.401	0.032
46	Phvul.003G202700	Nucleoside diphosphate phosphatase / Nucleoside-diphosphatase	DOR 364	261.399	0.456	0.169	0.043
47	Phvul.009G086400	Protein phosphatase 2c	DOR 364	1307.648	0.656	0.158	0.000
48	Phvul.002G223600	Inositol monophosphatase	DOR 364	179.400	1.696	0.395	0.000
49	Phvul.003G024900	8-oxo-dgtp diphosphatase / 8-oxo-dgtpase	DOR 364	497.180	1.091	0.267	0.001
50	Phvul.003G228700	Type i inositol 1/ 5-trisphosphate 5-phosphatase 2"	DOR 364	27672.527	1.090	0.292	0.002
51	Phvul.002G213400	Fructose-1/ 6-bisphosphatase-related	DOR 364	202.562	0.532	0.192	0.036

Six phosphate transporters genes were over expressed, three in IAC Imperador and three in DOR 364. Among them, two genes code for MSF transporters (Phvul.007G127400, Phvul.006G064900), the first up-expressed in IAC Imperador and the second in DOR 364. One up-expressed phosphate ABC transporter ATP-binding protein was found in IAC Imperador (Phvul.002G330700), which is responsible for coupling the energy of ATP hydrolysis to the import of phosphate across cellular membranes.

Finally, three genes with the SXP and EXS family domain were identified, Phvul.002G169700, Phvul.003G024600, and Phvul.007G275300, the first one up-expressed in IAC Imperador and the others in DOR 364.

In the enrichment test, 147 enriched gene ontologies (GO) were identified. From them, 123 were enriched for the IAC Imperador genotype under P restriction and 24 enriched for DOR 364, also under P restriction.

From the 123 enriched GOs in the IAC Imperador genotype, 52 represent sequences of the molecular function (FM) category and 71 represent the biological processes (BP) category. The terms of ontology were further divided into 123 functional subcategories. Within the MF category, most of the genes were observed in the following subcategories: catalytic activity (8%), binding (7%), organic cyclic compound binding (5%), heterocyclic compound binding (5%), ion binding (5%), small molecule binding (4%), nucleoside phosphate binding (4%), and nucleoside binding (4%). In the BP category, 71 subcategories were obtained, most of them in the following: metabolic process (13%), single-organism metabolic process (7%), response to stimulus (5%), small molecule metabolic process (4%), organonitrogen compound metabolic process (4%), phosphorus metabolic process (4%), phosphate-containing compound metabolic process (4%), response to stress (4%), oxoacid metabolic process (3%), and organic acid metabolic process (3%).

The genotype DOR 364 under P restriction exhibited 24 GOs terms, one classified as a cell component (CC), 15 as molecular functions (MF), and eight as biological processes (BP). The cell component subcategory exhibited nine gene sequences attributed to the codifying domains of the protein histidine kinase complex. The molecular function category exhibited 15 subcategories of gene ontology, with the most frequent codifying domains related to oxidoreductase activities (28%), ADP binding (13%), oxidoreductase activity acting on paired donors with incorporation or reduction of molecular oxygen and binding of iron ions (9%), iron ion binding (9%), tetrapyrrole binding (8%), heme binding (8%), monooxygenase activity (7%), molecular transducer activity (6%), and signal transducer activity (5%). In regard to the biological processes category, the unigenes were distributed in eight functional categories, in which the domains most frequently codify for the oxidation-reduction process (51%), defense response (34%), and signal transduction by phosphorylation system (7%).

Inside the BP category, our analysis showed the participation of 324 phosphorus metabolic process related genes. These genes are involved in chemical reactions and pathways involving P or P compounds, usually in the form of a phosphate group (PO4), anion, or salt of some phosphoric acid. All these genes were up-expressed in the IAC Imperador genotype under P restriction. Among these genes related to the P metabolic process, nine sequences were observed for the first time without homology with another gene already annotated.

#### Comparison 2. IAC Imperador vs. DOR 364 at the control P level

**Fig 2:**
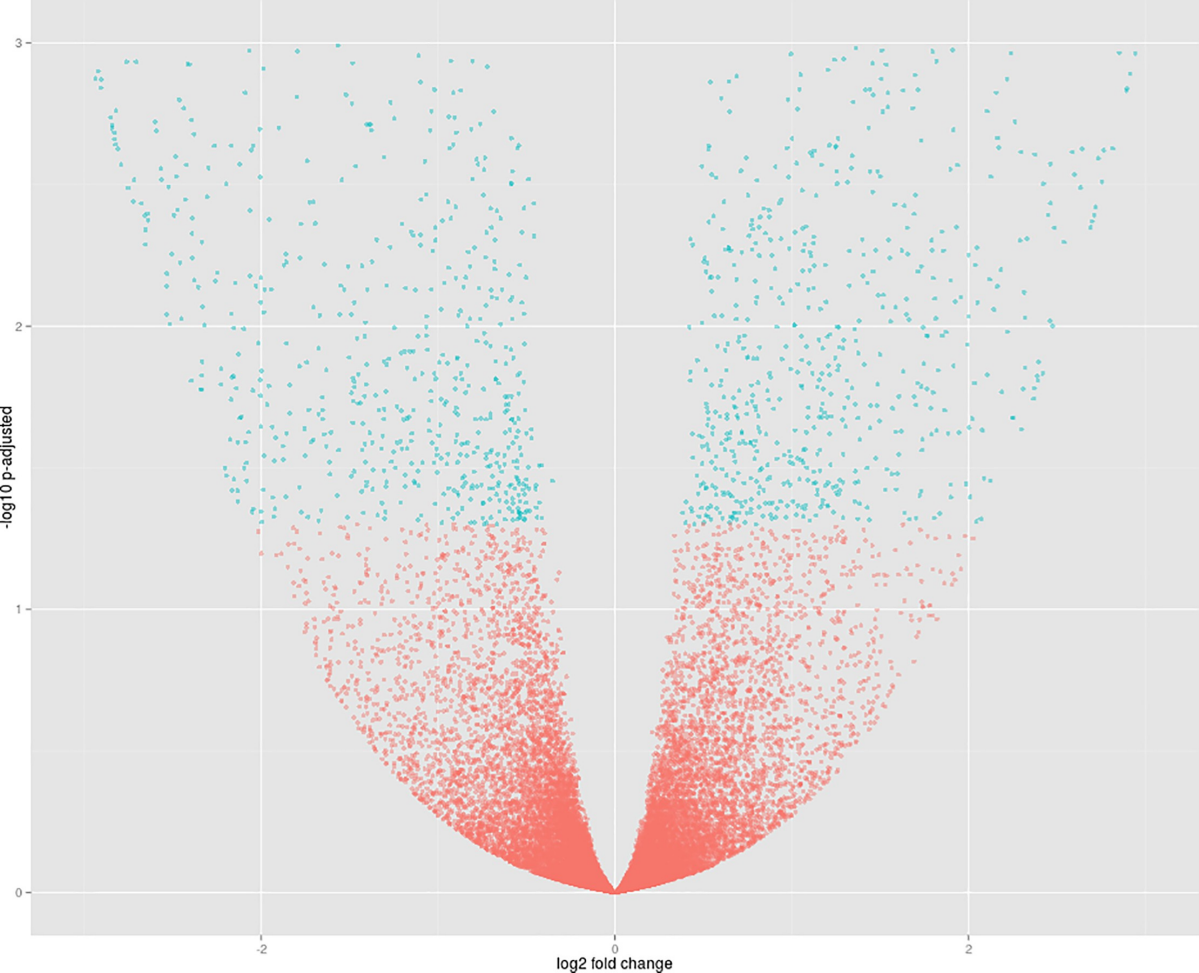
Volcano plot of comparison 2. Dots in blue represent significant differentially expressed genes. The genes of the IAC Imperador genotype (Libraries 7–9) are represented to the left of the origin of the coordinate plane, and the genes of DOR 364 (Libraries 10–12) to the right.

Considering libraries 7–12, IAC Imperador and DOR 364 in the control P level, 2161 differentially expressed genes were identified, in which 1069 genes were overexpressed in IAC Imperador and 1093 overexpressed in DOR 364 (Fig 2). In addition, 533 new genes were identified. It is also noteworthy that the number of differentially expressed genes is approximately half of the genes found in P restriction.

At the control P level, 49 transcription factors were identified, 21 being up-regulated in IAC Imperador and 27 in DOR 364. Ten up-regulated WRKY transcription factor families were found, eight in IAC Imperador and two in DOR 364. Five ethylene-responsive transcription factors were up-regulated in IAC Imperador, and two MYB transcriptional regulators were up-expressed in DOR 364. The response of the genotypes under the control P level in the up-regulation of transcription factors was about half of that expressed under P restriction.

Twenty-three up-expressed phosphatase genes were found, 11 in IAC Imperador and 12 in DOR 364. Among them, only three acid phosphatase up-regulated genes were found in DOR 364.

Only one up-regulated phosphate transporter gene was found in DOR 364 (Phvul.006G064900), which was identified as a sodium-dependent phosphate transporter.

Thirty-one ontology terms were observed in the IAC Imperador genotype, containing the molecular function and biological processes categories. The molecular function category was subdivided into 24 subcategories, and higher participation of genes was observed in relation to binding (11%), catalytic activity (11%), ion binding (8%), small molecule binding (5%), nucleoside phosphate binding (5%), nucleoside binding (5%), and anion binding (5%). In the biological processes category, only seven subcategories were observed, related to response to stimulus (36%), response to stress (26%), oxidation-reduction (18%), defense (15%), the flavonoid biosynthetic process (2%), the flavonoid metabolic process (2%), and the terpenoid catabolic process (1%).

Thirty-seven terms of gene ontology were assigned to the DOR 364 genotype under the control, and the enrichment test performed showed roles related to cell components, molecular functions, and biological processes, with higher participation of genes related to binding (11.54%), heterocyclic compound binding (8.84%), organic cyclic compound binding (8.84%), nucleoside phosphate binding (4.93%), and nucleotide binding (4.93%).

#### Comparison 3. IAC Imperador at the restrictive P level vs. IAC Imperador at the control P level

**Fig 3:**
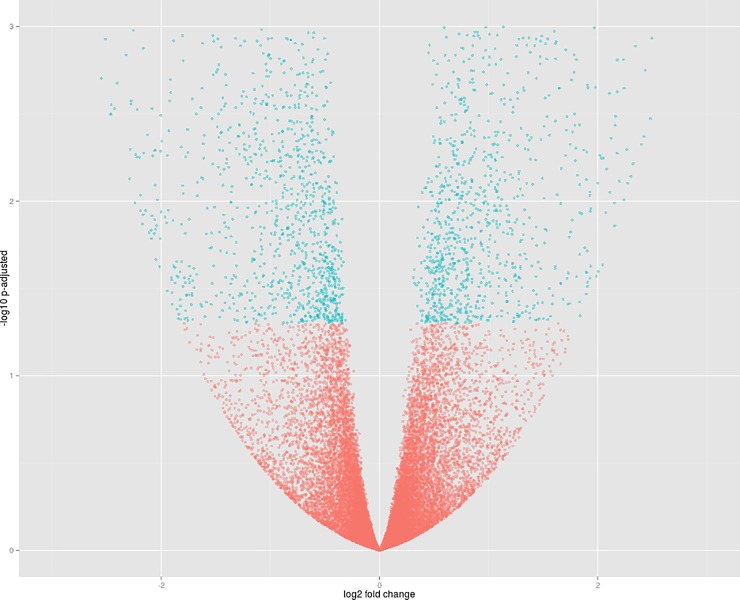
Volcano plot of comparison 3. Dots in blue represent significant differentially expressed genes. The genes of the IAC Imperador genotype under restrictive P (Libraries 1–3) are represented to the left of the origin of the coordinate plane, and the genes of the IAC Imperador genotype under control P (Libraries 7–9) to the right.

The libraries of IAC Imperador under P restriction (Libraries 1–3) and IAC Imperador under control (Libraries 7–9) treatments were compared, with identification of 3,217 differentially expressed genes, of which 1,537 were overexpressed in the nutrient restriction condition and 1680 under the control (Fig 3).

Among the 1538 genes differentially expressed under P restriction, the 25 most up-regulated and the 25 most down-regulated genes (Table 7) of greatest statistical significance were chosen as candidate genes in response to P deficit. Relative expression of the 25 most induced genes of the IAC Imperador genotype was from 10.6 to 33.2 times higher than under the control condition. In relation to the top-25 under-regulated genes, relative expression was from 9.9 to 44.5 times lower. In addition, 13 differentially expressed genes were not aligned, revealing new transcripts sequences related to stress from the P nutrient that can potentially be used for greater understanding of the functional responses of the genotype to P deficiency.

**Table 7:** Selection of the 25 candidate unigenes most up-regulated and the 25 candidate unigenes most down-regulated of IAC Imperador (decreasing order of log2FoldChange). From left to right: *gene id* (Phytozome); annotation of the gene based on *blastx search* using the database of P. vulgaris (Phytozome) with a cutoff of 1e-10; regulation; relative expression comparing restrictive P vs. control P level; baseMean; log2FoldChange; lfcSE (standard error); padj.

Nº	Unigene	Functional Annotation	Regulation	Relative Expression	baseMean	log2FoldChange	lfcSE	stat	padj
1	Phvul.007G091000	Hydroxyindole-O-methyltransferase and related SAM-dependent methyltransferases/protein dimerization activity	Up	33.249	88.154	5.055	0.421	11.998	0.000
2	Phvul.002G126200	Cytochrome P450 CYP2 subfamily/ oxidation-reduction process/electron carrier activity	Up	28.897	70.505	4.853	0.575	8.441	0.000
3	XLOC_002913	New sequence	Up	25.543	49.207	4.675	0.628	7.449	0.000
4	Phvul.005G162800	Not annotated	Up	24.253	56.416	4.600	0.467	9.856	0.000
5	XLOC_002909	New sequence	Up	23.380	45.320	4.547	0.612	7.427	0.000
6	Phvul.009G173900	Not annotated	Up	20.367	131.597	4.348	0.344	12.646	0.000
7	XLOC_013887	New sequence	Up	19.425	130.317	4.280	0.396	10.820	0.000
8	Phvul.004G032300	Calcineurin-like phosphoesterase/acid phosphatase related/hydrolase activity	Up	17.466	88.602	4.126	0.403	10.242	0.000
9	Phvul.003G144700	Not annotated	Up	15.730	340.916	3.975	0.363	10.951	0.000
10	Phvul.009G133000	Serine/threonine protein kinase/ATP binding/protein phosphorylation	Up	15.170	434.203	3.923	0.423	9.264	0.000
11	Phvul.002G065700	ALPHA-AMYLASE/cation binding	Up	14.573	165.394	3.865	0.528	7.317	0.000
12	XLOC_029866	New sequence	Up	13.624	26.165	3.768	0.597	6.316	0.000
13	Phvul.004G021300	Cytochrome P450 CYP2 subfamily/beta-carotene 15,15'-monooxygenase /oxidation-reduction process	Up	12.882	59.317	3.687	0.420	8.772	0.000
14	Phvul.002G003400	Cytochrome c oxidase, subunit vib/COX12/mitochondrion	Up	12.767	35.312	3.674	0.628	5.851	0.000
15	Phvul.008G045800	Vacuolar H+-atpase V1 sector, subunit G/vacuolar proton-transporting V-type atpase complex	Up	12.526	18.212	3.647	0.566	6.447	0.000
16	Phvul.011G169900	Trypsin and protease inhibitor endopeptidase inhibitor activity	Up	12.220	975.992	3.611	0.249	14.485	0.000
17	Phvul.006G143200	MBOAT, membrane-bound O-acyltransferase family	Up	12.018	42.499	3.587	0.479	7.484	0.000
18	XLOC_006513	New sequence	Up	11.878	15.996	3.570	0.653	5.468	0.000
19	Phvul.002G025000	Cytochrome P450 CYP2 subfamily/beta-carotene 15,15'-monooxygenase /oxidation-reduction process	Up	11.856	3423.318	3.568	0.276	12.935	0.000
20	Phvul.005G160800	Putative Phosphatase/Predicted haloacid dehalogenase-like hydrolase	Up	11.586	1552.905	3.534	0.336	10.526	0.000
21	Phvul.010G072000	Xenotropic and polytropic retrovirus receptor 1-related/spx (syg1/pho81/xpr1) domain-containing protein/Protein involved in vacuolar polyphosphate accumulation, contains SPX domain	Up	11.464	2086.707	3.519	0.396	8.896	0.000
22	Phvul.011G166500	Trypsin and protease inhibitor/endopeptidase inhibitor activity	Up	11.330	1295.436	3.502	0.230	15.253	0.000
23	Phvul.010G034400	Mlo protein/cell death/integral to membrane	Up	11.191	231.742	3.484	0.246	14.158	0.000
24	Phvul.007G249800	Calcineurin-like phosphoesterase/Predicted DNA repair exonuclease SIA1/hydrolase activity	Up	10.744	283.832	3.426	0.642	5.335	0.000
25	Phvul.004G099700	Leucine-rich repeat protein/protein binding	Up	10.651	20.278	3.413	0.580	5.886	0.000
26	Phvul.007G258500	Not annotated	Down	-44.563	43.720	-5.478	0.575	-9.529	0.000
27	Phvul.007G258600	Not annotated	Down	-44.563	43.720	-5.478	0.575	-9.529	0.000
28	Phvul.011G160700	Not annotated	Down	-41.063	173.434	-5.360	0.355	-15.087	0.000
29	Phvul.004G013500	Predicted carbonic anhydrase involved in protection against oxidative damage	Down	-36.974	45.463	-5.208	0.567	-9.189	0.000
30	XLOC_029764	New sequence	Down	-34.849	43.296	-5.123	0.571	-8.974	0.000
31	Phvul.009G075100	Branched chain aminotransferase BCAT1, pyridoxal phosphate enzymes type IV superfamily	Down	-20.455	2962.768	-4.354	0.329	-13.249	0.000
32	XLOC_019165	New sequence	Down	-18.631	16.058	-4.220	0.619	-6.812	0.000
33	XLOC_025262	New sequence	Down	-15.359	267.958	-3.941	0.626	-6.295	0.000
34	Phvul.006G087800	protein binding	Down	-14.156	482.425	-3.823	0.409	-9.337	0.000
35	Phvul.011G029200	Not annotated	Down	-13.742	126.533	-3.780	0.331	-11.419	0.000
36	Phvul.003G124300	Not annotated	Down	-13.161	72.624	-3.718	0.451	-8.237	0.000
37	XLOC_006364	New sequence	Down	-12.994	8239.221	-3.700	0.376	-9.840	0.000
38	Phvul.009G188800	Not annotated	Down	-12.918	21.557	-3.691	0.620	-5.954	0.000
39	Phvul.003G226900	Polysaccharide catabolic process/beta-amylase activity	Down	-12.493	1252.133	-3.643	0.303	-12.010	0.000
40	Phvul.010G165600	Serine/threonine-protein kinase	Down	-12.325	104.192	-3.624	0.344	-10.535	0.000
41	XLOC_016307	New sequence	Down	-11.741	29.163	-3.554	0.615	-5.774	0.000
42	Phvul.002G068500	Nucleobase-containing compound metabolic process/intracellular/3'-5' exonuclease activity/nucleic acid binding	Down	-11.348	62.755	-3.504	0.537	-6.523	0.000
43	XLOC_019294	New sequence	Down	-11.141	33.325	-3.478	0.508	-6.848	0.000
44	XLOC_019171	New sequence	Down	-11.119	12.963	-3.475	0.597	-5.825	0.000
45	Phvul.006G142700	Na+/dicarboxylate, Na+/tricarboxylate and phosphate transporters	Down	-11.032	2113.055	-3.464	0.292	-11.865	0.000
46	Phvul.008G282600	Raffinose synthase or seed imbibition protein Sip1	Down	-10.907	279.405	-3.447	0.243	-14.160	0.000
47	Phvul.001G085200	Light regulated protein Lir1	Down	-10.804	1449.564	-3.434	0.263	-13.049	0.000
48	Phvul.011G033800	heat shock protein binding	Down	-10.349	412.640	-3.371	0.368	-9.164	0.000
49	Phvul.003G274700	CTP synthase (UTP-ammonia lyase)	Down	-10.179	1060.325	-3.347	0.278	-12.052	0.000
50	XLOC_029515	New sequence	Down	-9.939	22.511	-3.313	0.587	-5.646	0.000

Under P deficiency, the IAC Imperador genotype enriched 118 functional subcategories, whereas under the control condition, 62 enriched subcategories were found. The main participants in enrichment in the P restriction response were genes related to metabolic processes (9.95%), catalytic activity (8.62%), the single-organism metabolic process (5.54%), transferase activity (3.44%), the small molecule metabolic process (3.15%), the phosphorus metabolic process (2.77%), the phosphate-containing compound metabolic process (2.75%), membrane parts (2.24%), the organic acid metabolic process (2.21%), and the carboxylic acid metabolic process (2.20%). Meanwhile, the main participants in enrichment in the control treatment were transition metal ion binding (7.10%), the response to chemical stimulus (6.60%), the nucleobase-containing compound biosynthetic process (5.84%), the response to abiotic stimulus (5.53%), and regulation of the nitrogen compound metabolic process (5.39%).

#### Comparison 4. DOR 364 at the restrictive P level vs. DOR 364 at the control P level

**Fig 4:**
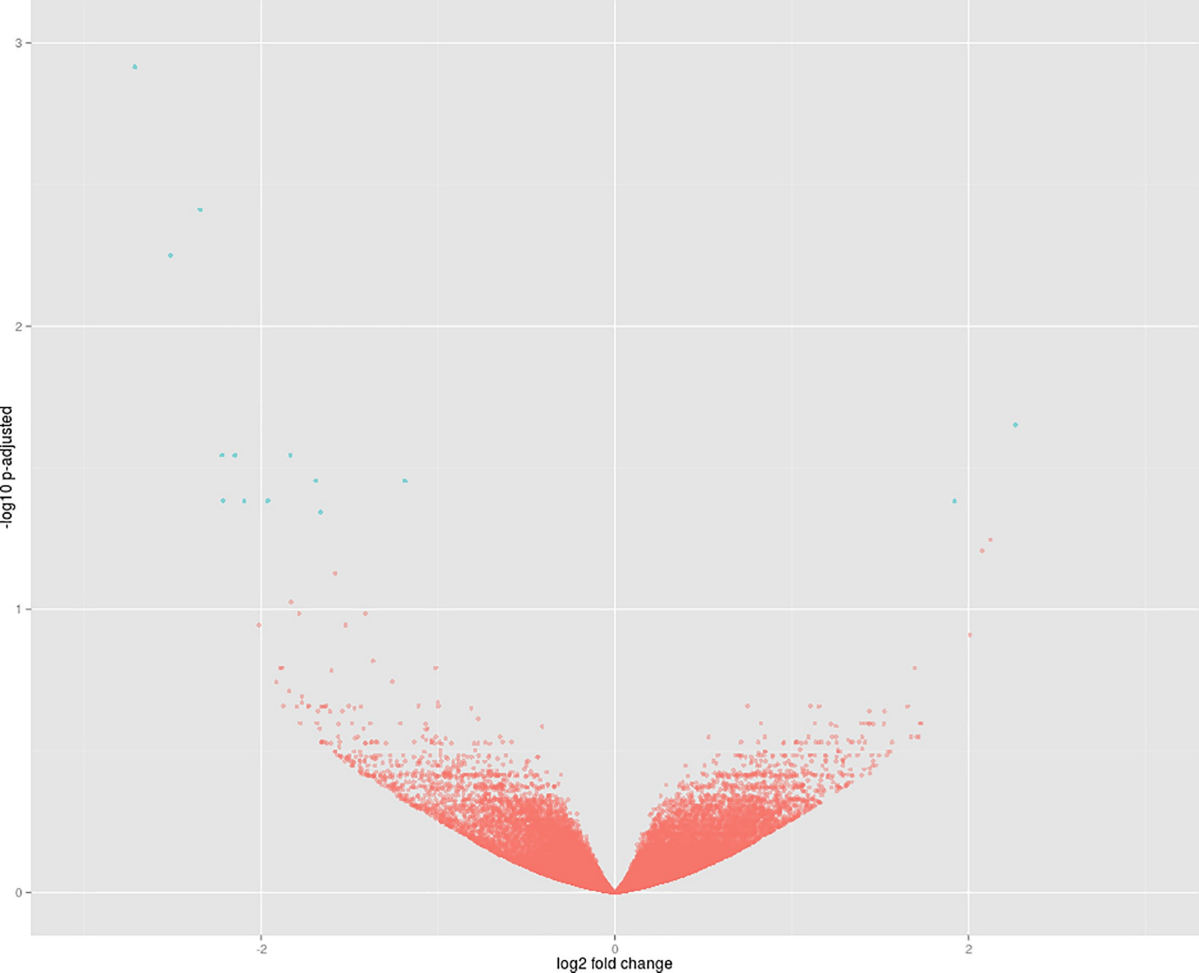
Volcano plot of comparison 4. Dots in blue represent significant differentially expressed genes. The genes of the DOR 364 genotype under restrictive P (Libraries 4–6) are represented to the right of the origin of the coordinate plane, and the genes of the DOR 364 genotype under control P (Libraries 10–12) to the left.

The libraries of DOR 364 under P restriction (Libraries 4–6) and under the control (Libraries 10–12) were compared, and, in contrast with the efficient genotype IAC Imperador, which exhibited 3217 differentially expressed genes, DOR 364 had only 15; two of them were up-regulated under P restriction and 13 up-regulated in the control (Table 8). The profile of genotype expression is shown in Fig 4.

**Table 8:** Unigenes induced from the DOR 364 genotype (increasing order of log2FoldChange). From left to right: *gene id* (Phytozome); annotation of the gene based on *blastx search* using the database of P. vulgaris (Phytozome) with a cutoff of 1e-10; regulation; relative expression comparing restrictive P vs. control P level; baseMean; log2FoldChange; lfcSE (standard error); padj.

N	Unigene	Functional Annotation	Regulation	Relative Expression	baseMean	log2FoldChange	lfcSE	stat	padj
1	Phvul.001G241600	Aminobenzoate/anthranilate synthase/ anthranilate synthase component /Isochorismate synthase	Up	4.810	12.652	2.266	0.494	4.583	0.022
2	Phvul.005G030100	Annexin/calcium ion binding/calcium-dependent phospholipid binding	UP	3.786	289.485	1.920	0.454	4.230	0.041
3	Phvul.010G010900	Cytochrome P450 CYP2 subfamily/oxidation-reduction process	Down	-2.982	173.066	-2.982	0.479	-6.225	0.000
4	Phvul.009G100300	Not annotated	Down	-2.714	15.997	-2.714	0.510	-5.327	0.001
5	XLOC_016307	New sequence	Down	-2.512	68.350	-2.512	0.512	-4.907	0.006
6	Phvul.006G139700	Not annotated	Down	-2.345	23.427	-2.345	0.466	-5.035	0.004
7	Phvul.010G062800	Domain of unknown function (DUF588)	Down	-2.222	54.550	-2.222	0.501	-4.440	0.029
8	Phvul.003G068700	Sequence-specific DNA binding transcription factor activity	Down	-2.216	10.346	-2.216	0.524	-4.226	0.041
9	Phvul.007G136800	Ga1, dna binding / calmodulin binding / transcription factor	Down	-2.149	172.980	-2.149	0.485	-4.431	0.029
10	Phvul.005G174400	ANION EXCHANGE PROTEIN/inorganic anion exchanger activity/membrane	Down	-2.097	20.695	-2.097	0.496	-4.229	0.041
11	Phvul.009G170600	CREB binding protein/P300 and related TAZ Zn-finger proteins/histone acetyltransferase activity	Down	-1.963	170.855	-1.963	0.460	-4.268	0.041
12	Phvul.009G137700	Multitransmembrane protein	Down	-1.835	169.636	-1.835	0.413	-4.440	0.029
13	Phvul.011G026300	Not annotated	Down	-1.692	97.664	-1.692	0.390	-4.337	0.035
14	Phvul.011G077900	Hydrolase activity, hydrolyzing O-glycosyl compounds/carbohydrate metabolic process	Down	-1.666	687.174	-1.666	0.398	-4.190	0.045
15	Phvul.007G229000	Not annotated	Down	-1.187	67.040	-1.187	0.273	-4.344	0.035

Relative expression of the two up-regulated genes observed in the DOR 364 genotype was 4.8 and 3.7 times greater under the P restriction condition than under the control condition, similar to relative expressions of the thirteen repressed genes (Table 8).

## Discussions

Analyses of variance show that the level of P applied influences development of all the shoot traits and seed yield. In addition, the root system traits did not show significant differences in accordance with P level. Thus, it can be inferred that even at low P concentrations, roots had the same level of development as the control rate.

A common response of plants to P deficiency is an increase in the size of the root system in relation to the shoots. The rate of formation of the shoots normally decreases with an increase in root growth, thus increasing the root/shoot ratio [10]. In this experiment, it was observed that plants grown under phosphorus restriction had a root/shoot ratio of 0.186, compared to 0.094 of plants grown under the control condition. IAC Imperador, the efficient and responsive genotype, had a ratio of 0.160, greater than the ratio of DOR 364, at 0.121.

In regard to the uptake, translocation, and P use indexes, DOR 364 had greater P concentrations in all plant tissues in comparison to IAC Imperador. In spite of exhibiting lower concentrations of P in its tissues, IAC Imperador had greater dry matter production, resulting in greater content of the element in the plant but consequent dilution of the nutrient. Considering the translocation efficiency index, although DOR 364 had a greater uptake index, IAC Imperador proved to translocate the element in the same manner, even with a smaller amount of P available in the plant tissue. In addition, IAC Imperador had a better efficiency index of P use in the shoots, total P use, and P use in the shoots for grain formation.

This greater efficiency and responsiveness of IAC Imperator can probably be explained by greater activation of the stress response genes and by greater dry matter production in relation to lower P concentration in the shoots under stress conditions. According to FAGERIA (1992) [11], the values of P use generally increase with a decrease in nutrient levels, and P use efficiency is maximized at the lowest nutrient level and minimized at the highest nutrient level.

According to SCHEIBLE AND ROJAS-TRIANA (2015) [12] plants respond to P limitation by changes in gene transcript expression, including genes involved in Pi transport, breakdown of P-containing molecules, photosynthesis, respiration, and amino acid and lipid metabolism. There are also changes in regulatory genes involved in signaling events, genes encoding transcriptional regulators (TRs) and microRNAs (miRs), components of the ubiquitin-proteasome system (UPS) for targeted protein degradation, and genes with functions that are yet unknown. As P-starvation develops gradually upon P withdrawal, P-starvation-responsive genes can be classified into early and late responsive groups. Early-responsive genes include many related to signal transduction events, general stress-related genes, genes encoding Pi transporters, SPX-domain proteins, purple acid phosphatases, ribonucleases, and also some cell wall-related genes. Late-responsive genes tend to be related to primary carbon metabolism, secondary metabolism, photosynthesis, protein synthesis, and hormone synthesis/signaling.

According to HIZ et al. (2014) [13], recent approaches to studying the profile of large-scale gene expression have proven to be an important tool for understanding how plants respond to biotic and abiotic stresses. The authors report that in spite of accumulation of information on transcriptome sequencing data of model plants and of agronomical important crops, few studies analyze the transcriptome under abiotic stress conditions in these species.

Under differential expression analysis, we found 4,123 differentially expressed genes under P restriction in comparison 1, almost double the 2,161 differentially expressed genes observed under the control P level in comparison 2. Regarding comparisons 3 and 4, within genotypes (restrictive P vs. control P), IAC Imperador, the P-responsive genotype, exhibited 3217 differentially expressed genes, whereas the P-unresponsive genotype, DOR 364, had only 15. Among the differentially expressed genes, some were identified above with P-starvation as transcription factors, phosphatase related genes, phosphate transporters, and sequences that were not aligned, i.e., those that revealed new transcripts related to P stress that can potentially be used for greater understanding of functional responses to P-deprivation.

Regarding the P restriction treatment, genes encoding proteins related to phosphorus restriction were identified, including P metabolism, phosphate-containing compound metabolic processes, nucleoside phosphate binding, phosphorylation, and response to stresses.

Macroarray analyses were performed by HERNÁNDEZ et al. (2007) [9] to evaluate gene expression from P-deficient roots of bean plants compared to control P-sufficient roots. They found 126 differentially expressed cDNAs, which were then compared by the BLASTX tool to the Uniprot protein database to assign putative function. Functional categories were as follows: 23% of genes were regulation/signal transduction; 23% were those coding for genes that participate in secondary metabolism pathways and/or were related to several stress/defense plant responses; 13% were classified as membrane proteins or proteins that participate in transport; 8% were classified in cell structure, cell cycle, or developmental functions; 24% were classified in different metabolic pathways, such as Pi cycling, C and N metabolism, amino acid/protein synthesis or degradation, and lipid metabolism; and 9% had no known function.

O'Rourke et al. (2012) [14] studied the transcriptome of White Lupin in regard to phosphorus deficiency and observed 904 differentially expressed genes in the roots, of which 396 were up-regulated in the P-sufficient treatment and 535 were up-regulated in the P-deficient treatment, i.e., greater up-regulation under P-restriction. In addition, the authors found 23 transcription factor families, with bHLH and AP2_EREB as the two largest families responding to the P deficiency stress identified.

OONO et al. (2013) [15] analyzed the differential expression profile of four rice genotypes with variation in growth response to P starvation, as indicated by the shoot/root dry weight ratio, also through RNA-seq. They identified differences in expression of transcripts under nutrient deficiency, observing 3,080 differentially expressed transcripts, in which most of the up-regulated transcripts were more strongly expressed in the tolerant genotypes, showing specificity in genotype response to P restriction. These differentially expressed transcripts include the NAC transcription factor, and it has been reported that NAC family mediated auxin and ethylene signaling promote lateral root development.

Considering the transcription factors, 101 differentially expressed genes were identified in the restrictive P level and 48 in the control P level. Among these genes, greater participation of the WRKY, ethylene-responsive element binding (ERF), and MYB families was observed.

According to SCHEIBLE AND ROJAS-TRIANA (2015) [12], reduction in the WRKY transcript halts gene expression responses, causing reduction in Pi uptake, early anthocyanin production, and increase in lateral roots and root-hair formation, regardless of P-status. WRKY regulators are also involved in negative regulation of phosphate by binding to the PHO1 promoter and by activating the Pi transporter in response to P-starvation.

We found 15 WRKY differentially expressed genes under P-starvation; 12 of them were up- regulated in IAC Imperador, i.e., 12 down-regulated and 3 up-regulated in DOR 364. According to CHEN et al. (2012) [16], several studies have indicated that WRKY transcription factors also participate in the nutrient deficiency response signaling pathways. AtWRKY75 was the first WRKY member reported to be involved in regulating phosphate starvation. It is strongly induced in the plant during Pi-deficiency and its suppression by RNAi silencing led to plants more susceptible to Pi stress, causing a decrease in Pi uptake and early accumulation of anthocyanin. In addition, the expression of several genes involved in Pi starvation responses, including phosphatases, Mt4/TPS1-like genes, and high-affinity Pi transporters, decreased when WRKY75 was suppressed.

The differential expression of 14 ethylene-responsive element binding factor (ERF) genes were found and 10 of them were up-expressed in IAC Imperador. The ERFs are known to be members of a novel family of transcription factors that are specific to plants [17]. ERF expression is up-regulated by various forms of biotic and abiotic stress, resulting in ethylene biosynthesis, and it is believed that ethylene might be a mediator of some stress responses.

Another type of differentially expressed transcription factor observed was MYB. The MYB transcription factor is arguably the most influential transcriptional regulator in the P-starvation response described in plants [12]. MYB also participates in metabolism, response to stress, and cell wall synthesis [18] (DALA VIA et al. 2015).

Five MYB differentially expressed transcripts were found. Four of them, Phvul.003G222900, Phvul.004G057800, Phvul.007G139300, and Phvul.007G211800, had repressed in IAC Imperador compared to DOR 364 under P deficiency.

RAMIREZ et al. (2013) [19] performed a comparative gene expression analysis through qRT-PCR of the regulatory genes that code for the MYB family TF: PvRHR1, Pv4, PvPHO2, and three isoforms of PvmiR399 from BAT477 and DOR364, classified as P-deficiency tolerant and P-deficiency sensitive common bean genotypes. According to the authors, PvRHR1 is a positive regulator of PvmiR399 and P-responsive genes, whereas Pv4 is a negative regulator of PvmiR399 by the target mimicry mechanism. PvPHO2 is a negative regulator of P-responsive proteins, and it is the target gene of PvmiR399. Their analysis, performed on roots, showed significant increases in the transcript levels of PvPHR1, Pv4, and PvmiR399 (isoforms a, b, and e) under P deprivation compared to the control in both genotypes. Furthermore, as expected, the transcript levels of the negative regulator PvPHO2 decreased 3.7-fold from BAT477 under P deficiency, though not in the P-sensitive genotype DOR364. However, DOR364 had a PvPHO2 transcript level that was 2.7 more in BAT477 under P deficiency.

According to GEORGE & RICHARDSON (2008) [5], the hydrolysis of organic P, a process necessary for Pi uptake by plant roots, is mediated by the action of phosphatase enzymes in the extracellular environment. This process of phosphatase activity is induced under P-deficiency conditions. LAN et al. (2015) also point out that acquisition of Pi is supported by the up-regulation of several intracellular and secreted Pi-releasing enzymes such as, pyrophosphorylases and other phosphatases of various types within the Pi starvation-inducible (PSI) core genes. In our analysis, 53 differentially expressed genes were found at the restrictive P level and 23 at the control P level, of which nine code for acid phosphatase, four for pyrophosphatase, and two for purple acid phosphatase.

In genome-wide associations (GWAs), ZHANG et al (2014) [20] identified a candidate gene *GmACP1* that encoded an acid phosphatase. Its over-expression in soybean hairy roots increased P efficiency by 11–20% compared to the control. A candidate-gene association analysis indicated that six natural *GmACP1* polymorphisms explained 33% of the phenotypic variation. The authors also conclude that the favorable alleles and haplotypes of *GmACP1* associated with increased transcript expression correlated with higher enzyme activity.

According to HERNÁNDEZ-DOMÍGUEZ (2012) [21], there are still a number of cellular events poorly characterized under low Pi conditions. This is the case of the processes involving pyrophosphate (PPi), a byproduct of the cellular metabolism of biosynthesis of nucleic acids, carbohydrates, proteins, and fatty acids. It is known that the soluble inorganic pyrophosphatases (PPase) recycle pyrophosphate, and may play a role in plant adaptation to phosphorus deficiency. Considering that, the authors measured PPase mRNA expression in leaves, stems, and roots of bean plants by qRT-PCR; their results reveal changes in the expression and activity of PPases under long-term Pi starvation, suggesting a possible role for PPi during plant adaptation to this condition. Such variations in expression and activity indicate the existence of individual regulatory mechanisms for each PPase isoform.

LIANG et al. (2012) [22] observed that throughout the period of P deficiency, the growth of both genotypes studied, G19833 (P-efficient genotype) and DOR364 (P-inefficient genotype), was inhibited after Pi starvation; whereas the internal acid phosphatase activities of both genotypes increased. The authors emphasize that the group of purple acid phosphatases (PvPAPs) plays a vital role in plant adaptive strategies to phosphorus deficiency, and it is known that PvPAPs can be classified into two groups, including a small group with low molecular weight, and a large group with high molecular weight. Analyses of qPCR expression showed that the large group, PvPAP1, was constitutively expressed in the roots, whereas PvPAP2 was specifically expressed in roots, but PvPAP2 expression levels in G19833 were close to those in DOR364. It is noteworthy that the transcripts of small PvPAPs dramatically increased during Pi starvation in both genotypes, and G19833 showed significantly more than those in DOR364.

Phosphate acquisition and distribution across plant tissues is also dependent on an array of transporters, which include proton-phosphate cotransporters belonging to the family of PHT proteins. Seven differentially expressed phosphate transporters were found; six were up-regulated at the restrictive P level and one at the control level. Among these transporters, two code for MSF transporters, one for the phosphate ABC transporter ATP-binding protein, three for SXP with EXS family domain, and one at the control P level for sodium-dependent phosphate transporters.

The major facilitator superfamily (MFS) transporter is known to facilitate transport across cytoplasmic or internal membranes from a variety of substrates, including ions, sugar phosphates, nucleosides, amino acids, and peptides. The SPX domain is found at the amino terminus of a variety of proteins. The PHO1 gene family conserved in plants is involved in a variety of processes, most notably the transport of inorganic phosphate from the root to the shoot of the plant and mediation of the response to low levels of inorganic phosphate, since the EXS family is involved in signaling transduction mechanisms [23].

According to GLASS and GIRVAN (2014) [24], enrichment tests for evaluating the functional properties of gene sets are a routine step in understanding high-throughput biological data and are used to verify that the genes implicated in a biological experiment are functionally relevant and to discover functions between those genes. Gene ontology (GO) is one of the most widely used functional enrichment tools in annotation of genes in different species.

In our enrichment test, 147 enriched GOs were identified under P restriction, while 71 GOs were found at the control P level. The GO terms associated with the unigenes were categorized in three main functional categories: cell components, molecular functions, and biological processes.

In relation to the treatment with P restriction, 21725 GO terms were associated and distributed at 0.04%, 59.97%, and 39.98% in the functional categories of cell components, molecular functions, and biological processes, respectively. In the control treatment, 7876 GO terms were associated; the molecular function category had the highest representation, at 89.02%, followed by the biological process category, at 10.74%, and, finally, the cell component category with a small share, at 0.22%. Under P restriction, the attribution of GO terms was 63.75% greater than under the control, with a bigger effective participation of the molecular function category in response to stress.

PATEL et al. (2014) [25] evaluated 20.4 million common bean unique reads from the NCBI database by the RNA-seq technique. Among these reads, 6,999 were mapped mapped on the common bean genome, and 1,679 known genes were identified, of which only 629 unigenes were annotated. In functional characterization, 3,724 terms of GO were attributed to the 629 annotated genes, which were distributed in the following manner: 46.37% molecular functions, 31.36% biological processes, and 22.26% cell components.

Differential expression analyses and enrichment test results corroborate the analyses and results observed regarding the efficiency indexes for P use, root/shoot ratio, grain yield, and genotype p-responsiveness under P restriction.

## Conclusions

Sequencing and analysis of the transcripts by the RNA-seq technique revealed differences in the gene expression profile between genotypes in both treatments, and confirmed the superiority of the IAC Imperador genotype in relation to DOR 364. The candidate genes related to P deprivation responses were able to be identified as transcriptor factors, phosphate transporters, and phosphatases. In addition, enrichment analysis showed that IAC Imperador enriched more subcategories than DOR 364, including P metabolic processes.

## Materials and methods

### Stress induction, analysis of agronomic traits, and estimation of efficiency in P uptake and use

The P-challenge experiment was conducted in a hydroponic greenhouse at the Instituto Agronômico de Campinas (Fazenda Santa Elisa, Campinas, SP, Brazil). The contrasting genotypes of common bean, IAC Imperador, previously classified as efficient and responsive, and DOR 364, as inefficient and non-responsive by SILVA et al. (2014) [7], were evaluated in regard to efficiency of P use.

A 2 × 2 factorial experimental design was applied, with the first factor constituted by two phosphorus application rates and the second factor constituted by the two genotypes. Nine replicates were used—three plants were collected in the full flowering stage (R6) to obtain gene expression profiles, three plants were collected to perform shoot and root system analysis, and three other replicates were grown until grain production.

The plants were grown in a closed hydroponic system in 3.5 L pots filled with medium particle size sand. The nutrient solution used in the two treatments was prepared with deionized water and was composed of 143.0 mg L^-1^ N, 132.5 mg L^-1^ K, 121.0 mg L^-1^ Ca, 25.5 mg L^-1^ Mg, 33.0 mg L^-1^ S, 1.81 mg L^-1^ Fe, 0.45 mg L^-1^ Cu, 0.18 mg L^-1^ Zn, 0.45 mg L^-1^ Mn, 0.45 mg L^-1^ B, 0.09 mg L^-1^ Mo, and 0.09 mg L^-1^ Ni. The treatments with P consisted of 4.00 and 8.00 mg L^-1^ P, constituting the restrictive P and control P levels, respectively. The plants were irrigated with a “half strength” solution, that is, with half the total concentration in the first two weeks after setting up the experiment. Electrical conductivity (1.8–2.0 mS cm^-1^) and pH of the nutrient solution (5.8) were checked daily and adjusted if necessary.

When the flowering stage (R6) was reached, three replicates were collected for evaluation of the following morphophysiological and agronomic traits: plant height (PH); number of nodes per plant (NNP); leaf area (LA); leaf, stem, and root dry matter (LDM, SDM, and RDM); root surface area (RSA), root diameter (RD), root length (RL), and root volume (RV). Chemical analyses of shoots, roots, and seeds were also performed. Three other replicates were collected to obtain the gene expression profile of the root system. Roots were softly washed with tap water to remove the sand and then immediately frozen in liquid nitrogen and stored in an ultra-freezer (-80°C).

For evaluation of morphophysiological traits, the three replicates were separated into shoots and roots. Total leaf area (cm^2^) was determined using an area meter (LI-COR, LI-3100C). These same plants were used for evaluation of shoot dry matter and were separated into leaves and stems and weighed on a precision balance. To obtain dry matter, the samples were dried in a forced air circulation laboratory oven at a temperature of 60°C.

After washing the root system for removal of the substrate, it was evaluated in regard to total length (cm), surface area (cm^2^), volume (cm^3^), diameter (mm), and dry matter (g). Images of the roots of each plant were made in a scanner (LA2400—EPSON), and then root characteristics were calculated through use of the WinRHIZO software (Regent Instruments Inc., Quebec, Canada).

Samples (1.0 g) of dry and ground plant tissue underwent nitric perchloric digestion to determine P concentration in the shoots, roots, and grains, which was determined by atomic absorption spectrophotometry [26].

The yield components evaluated were number of pods per plant (NPP), number of seeds per pod (NSP), 100 seed weight (100SW) in grams, and grain yield (GY) in g plant^-1^.

In accordance with the methodology proposed by SIDDIQI & GLASS (1981) [27] and modified by MOURA et al. (1999) [28], the following phosphorous-related indexes were estimated: P uptake efficiency (PUE), P translocation efficiency (PTE), P use efficiency in shoots (PUES), total P use efficiency (PUET), P use efficiency in shoots for grain dry matter production (PUEGP), and P harvest index (PHI).

The results of each variable were subjected to ANOVA and the F test in a 2 × 2 factorial design, and the mean values were compared by the Tukey test at 5% probability for quantification of the effects of the treatments.

### RNA-seq library preparation

The root systems of 12 samples, three biological replicates each of IAC Imperador and DOR 364 in the restrictive P and control P levels, were macerated, and approximately 100 mg were used for extraction of the total RNAs by the TRIzol Reagent extraction method [29]. The samples were sent to the Animal Biotechnology Laboratory of ESALQ/USP, where they were quantified in a Bioanalyser (Agilent) apparatus and adjusted to a concentration of 500 ng/μL. The libraries were prepared, using the TruSeq RNA Sample Prepv2 Low Throughput (LT) kit from Illumina according to manufacturer’s instructions, and sequenced with the Hiseq 2500 apparatus from Illumina.

### Pre-processing and data analysis

Demultiplexing was carried out using the CASAVA 1.8.2 (Illumina) software, which makes the base call of the raw data and transforms them into fastq format reads together with the phred quality scores. Reads quality was visualized using the FastQC program (www.bioinformatics.bbsrc.ac.uk/projects/). Filtering of low quality reads, sequences of adaptors, and vectors was performed by the Seqyclean v1.9.7 program (https://bitbucket.org/izhbannikov/seqyclean), using the 24 QScore. Contaminants were removed using the Univec database (http://www.ncbi.nlm.nih.gov/VecScreen/UniVec.html) as a reference. After filtering, reads with less than 65pb were removed.

### Mapping

The samples were mapped against the *Phaseolus vulgaris* genome (Pvulgaris 218 v1.0) on the Phytozome v.9.1 database. The Bowtie2 v2.1.0 [30] program was used for this step, selecting the ‘very-sensitive-local’ option. After that, mapping quality was checked for each sample using the Samtools v.0.1.18 package (FlagStat tool, LI et al., 2009). With the same program, mapping was organized (sort tool), and the file was used for obtaining isoforms through the Cufflinks 2.2.1 package [31]. In this step, assembly was obtained using the RABT option, along with the gtf annotation file available for *P*. *vulgaris* on Phytozome and the reference genome.

### Differential expression analysis

The files of the transcripts obtained for each sample were then compared using the Cuffmerge tool of the Cufflinks 2.2.1 package [31], in which the transcripts and isoforms are grouped into only one .gtf file. This file was used as the annotation offered to the script of the HTSeq-count v.05.4.p2 for extraction of raw read counts (http://www-huber.embl.de/users/anders/HTSeq/doc/index.html). After obtaining the counts, the groups were analyzed using the DESeq2 package [32] (LOVE et al., 2014) from R/Bioconductor [33]. Within the DESeq2 program, the data was normalized by library size. In order to avoid artifacts caused by low expression profiles and high expression variance, only transcripts that had an average baseMean > 5 and the mean greater than the standard variation were analyzed. After that, the normalized counts from transcripts that passed in filtering were analyzed using the generalized linear model (GLM). The Benjamini-Hochberg [34] (BENJAMINI & HOCHBERG, 1995) False Discovery Rate (FDR) correction for multiple tests was applied.

To better understand the response mechanisms among the P levels applied and the genotypes, four different comparisons of differential expression tests were performed. First, genotypes were compared within each P rate applied as follows: Comparison 1. IAC Imperador vs. DOR 364 at the restrictive P level; and Comparison 2. IAC Imperador vs. DOR 364 at the control P level. Second, each genotype response was compared in accordance with the different P levels applied as follows: Comparison 3. IAC Imperador at the restrictive P level vs. IAC Imperador at the control P level; and 4. DOR 364 at the restrictive P level vs DOR 364 at the control P level.

The transcripts obtained from the Cufflinks merged .gtf file were aligned against a selection from the non-redundant NCBI database (nr, date: Nov. 12, 2013) using the Viridiplantae based protein subgroup (tax id 33090, August 2014). The blastx program with the 1e-10 cutoff was used [35, 36] (ALTSCHUL et al., 1990; CAMACHO et al., 2009). Functional annotation of the terms of gene ontology (GO) and its derivatives was carried out using the Blast2GO—b2g4pipe v2.5 program [37] (CONESA et al., 2005), with 20 hits of Blast for each contig. Enrichment analysis was performed by B2GO using the Fisher exact test and considering only the categories with FDR <0.05. The genes that passed through the filtering of the baseMean >5 of each analysis of differential expression were used as background.

## References

[1] Miklas PN, Kelly JD, Beebe SE, Blair MW. Common bean breeding for resistance against biotic and abiotic stresses: From classical to MAS breeding. Euphytica. 2006; 147:1–2.

[2] Westermann D, Terán H, Muñoz-Perea C, Singh S. Plant and seed nutrient uptake in common bean in seven organic and conventional production systems. *Canadian Journal of Plant Science*.2011; 91: 1089–1099. 10.4141/cjps10114

[3] Raghothama K G. Phosphate Acquisition. Annual Review of Plant Physiology and Plant Molecular Biology 1999; 50: 665–693. 10.1146/annurev.arplant.50.1.665 15012223

[4] Mourice S,K, Tryphone G M. Evaluation of Common Bean (Phaseolus vulgaris L.) Genotypes for Adaptation to Low Phosphorus. ISRN Agronomy. 2012 2012: 1–9. 10.5402/2012/309614

[5] George T S, Richardson A E. Potential and limitations to improving crops for enhanced phosphorus utilization IN: White PJ, Hammond JP. The ecophysiology of plant-phosphorus interactions. Springer 2008; 247–270.

[6] White P J, Hammond J P. Phosphorus Nutrition Of Terrestrial Plants, 51–81 In: White PJ, Hammond JP. The ecophysiology of plant-phosphorus interactions. Vol. 7 Dordrecht, the Netherlands:Plant Ecophysiology. Springer 2008; pp Springer. 2008; pp 51–81.

[7] Silva DA, Esteves J A, F, Messias U, Teixeira A, Gonçalves JG R, Chiorato AF, et al Efficiency in the use of phosphorus by common bean genotypes. Scientia Agricola. 2014;71:3, 232–239.

[8] Fageria NK, Carvalho MCS, Knupp AM Phosphorus Nutrition of Upland Rice, Dry Bean, Soybean, and Corn Grown on an Oxisol. Communications in Soil Science and Plant Analysis. 2015; 46:9 10.1080/00103624.2015.1019083

[9] Hernández G, Ramírez M, Valdés-López O, Tesfaye M, Graham M A, et al Phosphorus stress in common bean: root transcript and metabolic responses. Plant Physiology. 2007; 144:2, 752–67. 10.1104/pp.107.096958 17449651 PMC1914166

[10] Lynch J P, Brown K M. Root strategies for phosphorus acquisition IN: White PJ, Hammond JP. The ecophysiology of plant-phosphorus interactions. Vol. 7 Dordrecht, the Netherlands:Plant Ecophysiology. Springer 2008; pp 83–116

[11] Fageria NK. Maximizing crop yields New York: Marcel Dekker 1992; 274p.

[12] Scheible WR, Rojas-Triana M. Sensing, signalling, and control of phosphate starvation in plants: Molecular players and applications. In Annual Plant Reviews Phosphorus Metabolism in Plants. 2015; 48: 25–64. 10.1002/9781118958841.ch2

[13] Hiz MC, Canher B, Niron H, Turet M. Transcriptome analysis of salt tolerant common bean (Phaseolus vulgaris L.) under saline conditions. *PloS One*. 2014; 9:3 10.1371/journal.pone.0092598 PMC3961409 24651267

[14] O'rourke JA, Yang SS, Miller SS, Bucciarelli B, Liu J, Rydeen A, et al An RNA-Seq Transcriptome Analysis of Pi Deficient White Lupin Reveals Novel Insights Into Phosphorus Acclimation in Plants. Plant Physiology.2012; 161:705–724. 10.1104/pp.112.209254 23197803 PMC3561014

[15] Oono Y, Kawahara Y, Yazawa T, Kanamori H, Kuramata M, Yamagata H, et al Diversity in the complexity of phosphate starvation transcriptomes among rice cultivars based on RNA-Seq profiles. Plant Molecular Biology.2013; 83: 523–537. 10.1007/s11103-013-0106-4 23857470 PMC3830200

[16] Chen L, Song Y, Li S, Zhang L, Zou C, Yu D. The role of WRKY transcription factors in plant abiotic stresses. Biochimica et Biophysica Acta. 2012; 1819: 120–128. 10.1016/j.bbagrm.2011.09.002 21964328

[17] Fujimoto SY, Ohta M, Usui A, Shinshi H, Ohme-Takagi M. Arabidopsis Ethylene-Responsive Element Binding Factors Act as Transcriptional Activators or Repressors of GCC Box—Mediated Gene Expression. 2000;12: 393–404. 10715325 10.1105/tpc.12.3.393 PMC139839

[18] Dalla Via V, Narduzzi C, Aguilar OM, Zanetti ME, Blanco FA. Changes in the common bean transcriptome in response to secreted and surface signal molecules of Rhizobium etli. Plant Physiology. 2015; 169:2,1356–1370. 10.1104/pp.15.00508 26282238 PMC4587446

[19] Ramirez M, Flores-Pacheco G, Reyes JL, Álvarez A L, Drevon J J, Girard L, et al Two common bean genotypes with contrasting response to phosphorus deficiency show variations in the microRNA 399-mediated PvPHO2 regulation within the PvPHR1 signaling pathway. Int J Mol Sci. 2013; 14(4): 8328–8344. 10.3390/ijms14048328 23591845 PMC3645745

[20] Zhang D, Song H, Cheng H, Hao D, Wang H, Kan G, et al The acid phosphatase-encoding gene GmACP1 contributes to soybean tolerance to low-phosphorus stress. PLoS Genet. 2014; 10:1 10.1371/journal.pgen.1004061 PMC3879153 24391523

[21] Hernández-Domíguez EE, Valencia-Turcotte L G, Rodríguez-Sotres R. Changes in expression of soluble inorganic pyrophosphatases of Phaseolus vulgaris under phosphate starvation. Plant Science.2012; 187: 39–48. 10.1016/j.plantsci.2012.01.009 22404831

[22] Liang C, Sun L, Yao Z, Liao H, Tian J. Comparative analysis of gene family and their functions in response to phosphorus deficiency PVPAP in common bean. PLoS ONE.2012;7:5 10.1371/journal.pone.0038106 PMC3360649 22662274

[23] Poirier Y, Jung JY. Phosphate TransportersIn: Annual Plant Reviews Volume 48: Phosphorus Metabolism in Plants, First Edition Edited by Plaxton William C. and Lambers Hans. John Wiley & Sons, Ltd 2015 10.1002/9781118958841.ch5.

[24] Glass K, Girvan M. Annotation Enrichment Analysis: An Alternative Method for Evaluating the Functional Properties of Gene Sets. Scientific reports.2014; 4: 4191 10.1038/srep04191 24569707 PMC3935204

[25] Patel SS, Shah DB, Panchal HJ. De Novo RNA Seq Assembly and Annotation of Phaseolus vulgaris L. (SRR1283084). Genomics and Applied Biology. 2014; 5:5, 1–6.

[26] Malavolta E, Vitti GC, Oliveira AS. Avaliação do estado nutricional das plantas: princípios e aplicações. Piracicaba: Associação Brasileira para Pesquisa do Potassio e do Fosfato. p. 201, 1989.

[27] Siddiqi M Y, Glass D M. Use index: a modified approach to the estimation and comparison of nutrient use efficiency in plants. Journal of Plant Nutrition, New York. 1981; 4: 289–302.

[28] Moura W M, Casali VWD, Cruz CD, Lima PC. Divergência genética em linhagens de pimentão em relação à eficiência nutricional de fósforo. Pesquisa Agropecuária Brasileira, Brasília.1999;34:2.

[29] Chomczynski P, Sacchi N. Single Step Method of RNA Isolation by Acid Guanidinium Thiocyanate-Phenol-Chloroform Extraction. Anal. Biochem.1987; 162:156–159 10.1006/abio.1987.9999 2440339

[30] Langmead B, Salzberg S. Fast gapped-read alignment with Bowtie 2. Nature Methods. 2012; 9:357–359. 10.1038/nmeth.1923 22388286 PMC3322381

[31] Trapnell C, Williams BA, Pertea G, Mortazavi AM, Kwan G, Van Baren MJ, et al Transcript assembly and quantification by RNA-Seq reveals unannotated transcripts and isoform switching during cell differentiation.Nature Biotechnology.2010; 28: 511–515. 10.1038/nbt.1621 20436464 PMC3146043

[32] Love MI, Huber W, Anders S. 2014 Moderated estimation of fold change and dispersion for RNA-Seq data with DESeq2. bioRxiv. Genome Biol. 2014; 15(12):550 10.1101/002832, 10.1101/002832. 25516281 PMC4302049

[33] Gentleman R, Carey VJ, Bates DM, Bolstad B, Dettling M, Dudoit S, et al Bioconductor: Open software development for computational biology and bioinformatics R. Genome Biology. 2004;5:80. 10.1186/gb-2004-5-10-r80 PMC545600 15461798

[34] Benjamini Y, Hochberg Y. Controlling the false discovery rate: a practical and powerful approach to multiple testing. Journal of the Royal Statistical Society, Series B (Methodological). 1995; 57:289–300.

[35] Altschul SF, Gish W, Miller W, Myers EW, Lipman DJ. Basic local alignment search tool. J MolEvol.1990;215: 403–410. 10.1016/S0022-2836(05)80360-2 2231712

[36] Camacho C, Coulouris G, Avagyan V, Ma N, Papadopoulos J, Bealer K, et al BLAST+: architecture and applications. BMC Bioinformatics. 2009; 10:421 10.1186/1471-2105-10-421 20003500 PMC2803857

[37] Conesa A, Gotz S, Garcia-Gomez JM, Terol J, Talon M, Robles M. Blast2GO: a universal tool for annotation, visualization and analysis in functional genomics research. Bioinformatics.2015; 21:3674–3676. 10.1093/bioinformatics/bti610 16081474

